# One-step synthesis of red-emitting carbon dots *via* a solvothermal method and its application in the detection of methylene blue†

**DOI:** 10.1039/c9ra05570c

**Published:** 2019-09-18

**Authors:** Dan Zhao, Xuemei Liu, Chunjin Wei, Yimo Qu, Xincai Xiao, Han Cheng

**Affiliations:** School of Pharmaceutical Science, South-Central University for Nationalities Wuhan 430074 P. R. China

## Abstract

The synthesis of carbon dots (CDs) with long wavelengths, particularly the red-emitting ones, has always been the focus of researchers, and a carbon source is critical in this process. In this study, we report the synthesis of red-emitting CDs (CD-tetra) *via* a one-step solvothermal method with 1,2,4,5-benzenetetramine tetrahydrochloride as a novel carbon source and ethanol as a solvent, and the quantum yield (QY) of CDs is as high as 30.2%. Middle chromatography isolated gel (MCI Gel) column was used to obtain R-CDs, O-CDs and Y-CDs with emission wavelengths at 619, 608 and 554 nm, respectively. It was discovered that these CDs exhibited great differences in their particle sizes and elemental compositions. Moreover, the fluorescence of the CD-tetra could be efficiently quenched using methylene blue (MB). Under optimal conditions, a linear relationship between the decreased fluorescence intensity of the CD-tetra and the concentration of MB was established in the range of 0.05–9.5 μM. The limit of detection (LOD) is 10 nM, suggesting a promising assay for the detection of MB.

## Abstract 2

Red-emitting CDs was synthesized *via* a one-step solvothermal method with 1,2,4,5-benzenetetramine tetrahydrochloride as a novel carbon source and ethanol as a solvent. The luminescence mechanism of CDs was studied by MCI gel column chromatography.

## Graphical abstract

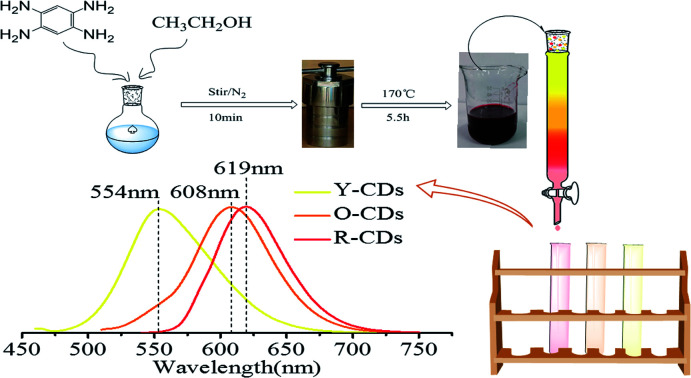

## 1. Introduction

CDs, with their fluorescence capability, are an attractive, carbon-based, functional materials after the appearance of graphene, carbon nanotubes and nanodiamonds,^1^ and they are widely used in various fields due to their multiple preparation routes, low toxicity, excellent biocompatibility and chemical stability.^2^ In recent years, the long-wavelength emitting CDs have been the focus of researchers because of their promising potential in cell imaging,^3–5^*in vivo* imaging,^6,7^ and *in vivo* labeling and diagnosis.^8,9^ The reports and papers on green, yellow and orange emitting CDs^10–12^ have been widely seen in published journals, but the ones on red-emitting CDs^13^ are relatively scarce.

The selection of proper raw materials is the critical factor that causes the difficulty in the preparation of red-emitting CDs. At present, the raw materials for preparing the red-emitting CDs are mainly two types; one of which is an aromatic compound. Xiong *et al.*^14^ prepared red-emitting CDs (*λ*_em_ at 640 and 715 nm) with *O*-phenylenediamine and glutamic acid as the raw materials, and sulfuric acid as the solvent. This method required a high reaction temperature (210 °C) and a long experimental time (10 h). Zhang *et al.*^15^ used phenylenediamine as the raw material and toluene as the solvent to prepare red-emitting CDs. The high toxicity of the solvent toluene greatly limits its expansion for practical applications. The second type is material by doping sulfur/phosphorus. Miao *et al.*^16^ prepared red-emitting N, S-CDs *via* the solvothermal method with citric acid (CA) as the carbon source and thiourea as the sulfur dopant. The maximum emission peak of the as-prepared CDs was at 594 nm. Hu *et al.*^17^ mixed CA or ethylene glycol (EG) with ethylenediamine polyethylenimine, and added H_3_PO_4_ to prepare the red-emitting CDs. However, the use of multiple types of raw materials, increased variables and complicated synthesis process made this preparation method difficult. The one-step synthesis exhibited its merits (*e.g.*, low-cost and simple process). The solvothermal method is the most advantageous one in preparing the red-emitting CDs.^18^ Based on this, a new aniline compound was selected as the raw material to realize the fast and simple preparation of red-emitting CDs.

It is worth mentioning that in addition to the preparation, the luminescence mechanism is also an area of focus in CDs research. The multiple synthesis methods for CDs make it difficult to study the synthesis process, and also hinder the progress on the study of their luminescence mechanism. The direct characterization of CDs *via* their acquired products has been reported in most published literature.^19,20^ Several researchers have reported their studies on the luminescence mechanism based on the separation of the acquired products.^21,22^ Ding *et al.*^21^ used silica column chromatography to separate the product and acquired CDs with emission wavelengths ranging from 440 nm to 625 nm, and finally proved that the red-shifting of the emission peak was the result of the increase in the degree of surface oxidation.^21^

Although several high separation efficiency techniques have been reported, these methods exhibit obvious drawbacks. For example, high performance liquid chromatography (HPLC)^23,24^ and polyacrylamide gel electrophoresis (PAGE)^25^ require repeated separations to acquire sufficient amounts, and in addition, the separation effect of SiO_2_ chromatography is not good and the loss is large. In this study, we report the application of MCI Gel as a filler to realize CDs product separation. The MCI Gel is a polystyrene-based, reversed-phase resin filler exhibiting excellent separation efficiency with a large loading amount.

Methylene blue (MB) is a thiazide dye and is widely used as a near-infrared dye. High concentrations of MB can poison animals and cause death. The residues of MB in aquatic products have also received attention, and studies have found that such dyes and metabolites are teratogenic.^26,27^ It is necessary to detect the content of methylene blue. At present, methods for detecting MB include early spectrophotometry,^28^ liquid chromatography^29,30^ and capillary electrophoresis.^31^ Some of these methods have lower sensitivity and are unsuitable for microanalysis. In addition, some operations can be cumbersome, whereas the spectrophotometric method is simple and rapid, so it is suitable for detecting MB.

This paper reports the preparation of red-emitting CD-tetra *via* a fast and simple one-step solvothermal route with 1,2,4,5-benzenetetramine tetrahydrochloride as an original carbon source. The QY of the as-prepared CDs is up to 30.2%, and the as-prepared CDs exhibit an excitation wavelength dependent property. To better understand the luminescence mechanisms, the MCI Gel column chromatography was selected to realize the separation of the synthesis product, and the red, orange and yellow-emitting, excitation wavelength independent CDs were finally acquired. Based on these experiments, a reasonable synthesis and luminescence mechanism are proposed. In addition, the fluorescence of the as-prepared CDs is quenched in the presence of MB. Therefore, the as-prepared CDs may work as fluorescent probes for the rapid detection of MB.

## 2. Experimental section

### 2.1 Reagents and materials

1,2,4,5-Benzenetetramine tetrahydrochloride (AR, 95%) was acquired from Adamas Reagent Co., Ltd. 1,2,4-Benzenetriamine dihydrochloride (AR, 97%) was purchased from Saan Chemical Technology (Shanghai) Co., Ltd. *O*-Phenylenediamine (AR, 98%) was obtained from Aladdin Industrial Corporation. Solvents were purchased from Sinopharm Chemical Reagent Co., Ltd., all of which were of AR grade (unless otherwise stated). The MCI Gel filler (CHP20/P120) was purchased from the Mitsubishi Chemical Corporation of Japan, and Rhodamine 6G and Rhodamine B were purchased from Aladdin Chemistry Co., Ltd. NaH_2_PO_4_, Na_2_HPO_4_, methylene blue, Sudan I (SDI), methyl red (MR), CoCl_2_, ZnCl_2_, FeCl_3_, CaCl_2_, CuCl_2_, CdCl_2_ and HgCl_2_ were purchased from Sinopharm Chemical Reagent Co., Ltd. (Shanghai, China). Deionized (DI) water was used throughout the experiment.

### 2.2 Characterization

A LS55 fluorescence spectrophotometer (PerkinElmer) was used to record the fluorescence spectra, and a Lambda-35 UV-Vis spectrophotometer (PerkinElmer) was used to measure the UV-Vis absorption spectra. A Nicolet 6700 Fourier infrared spectrometer (Malvern, UK) was used to record FTIR spectra. A VG Multilab 2000 X-ray photoelectron spectroscopy instrument was used to perform surface analyses and a JEM-2100F transmission electron microscope (Japan Electron Optics Laboratory Company) was used to characterize the surface morphology of the as-prepared CDs. The pH was monitored by a PHSJ-3F pH meter (Shanghai Precision Scientific Instrument Company). X-ray power diffraction (XRD) patterns were recorded on a D8-Advance spectrometer (Bruker, Germany).

### 2.3 Synthesis of CDs

CDs were synthesized in one step by the solvothermal method. The synthesis procedure is as follows: 0.025 g of 1,2,4-benzenetriamine dihydrochloride powder was dissolved in 24 mL of ethanol and stirred well for 10 min with N_2_. The mixture was then transferred into a 25 mL Teflon-lined stainless-steel autoclave and kept at 170 °C for 5.5 h. Subsequently, the reaction solution was cooled down. The same reaction conditions and steps were used as described above for *O*-phenylenediamine and 1,2,4-benzenetriamine dihydrochloride.

### 2.4 Quantum yield (QY) measurements

Rhodamine 6G (QY = 95%) and Rhodamine B (QY = 56%) dissolved in absolute ethanol (refractive index *η* = 1.096, 25 °C) were used as references.^32^ The QY of the CDs was calculated by comparing the ratio of the fluorescence area (Rhodamine 6G-*λ*_ex_ = 488 nm and Rhodamine B-*λ*_ex_ = 495 nm) to the absorbance. All samples were dissolved in absolute ethanol and their absorbance at 488 nm or 495 nm was controlled to be less than 0.1. The relative QY is calculated using the following equation:*Φ*_X_ = *Φ*_ST_(*G*_rad_X/*G*_rad_ST)(*η*_X_^2^/*η*_ST_^2^)where *Φ* is QY, *G*_rad_ is the ratio of the fluorescence area to the absorbance, *η* is the refractive index of the solvent, ST is the standard substance, and X is the CDs sample, where *η*_X_ = *η*_ST_.

### 2.5 Detection of MB

CD-tetra, ethanol and the different detected samples were made up to a final volume of 1 mL, followed by the addition of the MB standard at various concentrations. The fluorescence emission spectra were recorded after a reaction time of 1 min. The selectivity of the MB sensing was confirmed by adding common metal ion stock solutions (including Co^2+^, Zn^2+^, Fe^3+^, Ca^2+^, Cu^2+^, Cd^2+^, and Hg^2+^ ions) and other dyes (Sudan I and MR) instead of MB and replicating the experimental procedure. The fluorescence was recorded at an excitation wavelength of 540 nm. All experiments were performed at room temperature.

## 3. Results and discussion

### 3.1 Synthesis of red-emitting CD-tetra

#### 3.1.1 The effects of the number of amino groups on the anilines

**Fig. 1:**
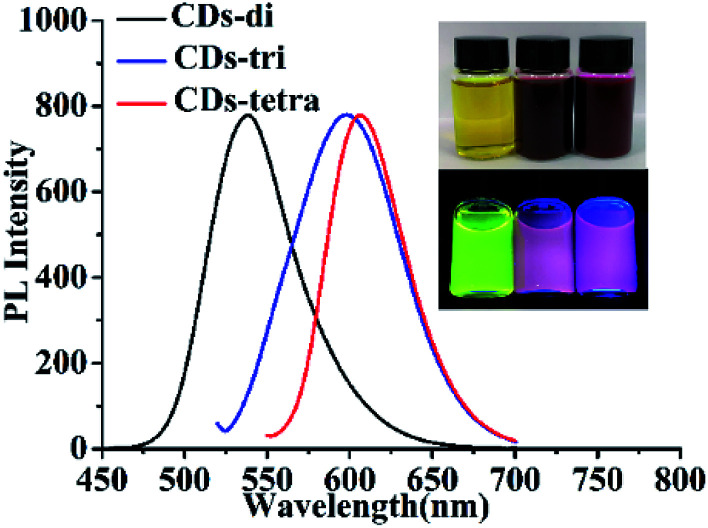
Fluorescence spectra of CD-di, CD-tri and CD-tetra. Inset: Ambient lighting (top) and 365 nm UV lamp (bottom) with CD-di, CD-tri and CD-tetra solutions from left to right.

The type of raw material plays a critical role in the CD synthesis. The impact of the number of amino groups on the aniline derivatives upon the as-prepared CDs was investigated. With the other reaction parameters fixed (24 mL absolute ethanol, 170 °C and 5.5 h), three anilines (*O*-phenylenediamine, 1,2,4-benzenetriamine dihydrochloride and 1,2,4,5-benzenetetramine tetrahydrochloride, 0.025 g) were used to prepare the CDs. The CDs prepared with *O*-phenylenediamine, 1,2,4-benzenetriamine dihydrochloride and 1,2,4,5-benzenetetramine tetrahydrochloride were named CDs-di, CDs-tri and CDs-tetra, respectively. The excitation and emission wavelengths, QYs and references of the as-prepared CDs are shown in Table 1, and the related calculations for QYs shown in the ESI.† The QYs of the three CDs were measured in ethanol (the QY of CD-tetra reached 30.2% (Fig. S1†)). In addition, the fluorescence spectrum of the three CDs are shown in Fig. 1, and the photographs of the CD-di, CD-tri and CD-tetra solutions in ambient light (top) and 365 nm UV light (bottom) are shown in the inset (from left to right). With the increased number of amino groups, the excitation and emission wavelengths all exhibited a redshift. Although the emission wavelengths of CD-tri and CD-tetra do not differ by much, the QY of CD-tetra greatly increases, showing that the amino group number plays a critical role in the synthesis process. We speculate that the greater number of amino groups (the active group) on the benzene ring would ensure a large number of active sites during the conjugation reaction, which would be more beneficial to the synthesis of CDs with larger particle sizes and rich nitrogen content. Since the CD-tetra exhibited excellent optical properties, it was thus chosen as the target for further research.

**Table 1:** The excitation wavelengths, emission wavelengths, QYs and reference of CD-di, CD-tri and CD-tetra

Sample	*λ*_ex_/nm	*λ*_em_/nm	QY	Reference
CDs-di	420	538	16.7%	Rhodamine 6G
CDs-tri	510	598	13.4%	Rhodamine B
CDs-tetra	540	605	30.2%	Rhodamine B

#### 3.1.2 The impact of the amount of raw materials

The amount of raw materials also has an impact on the optical properties of the as-prepared CDs, and it was thus investigated. With other experimental parameters fixed, as shown in Fig. S2,† the increase in the raw material amount (from 0.005 g to 0.025 g) led to a dramatic increase in the fluorescence intensity of the asprepared CDs. Interestingly, the amount of raw materials had a great influence on the fluorescence color and intensity of the CDs, but had little effect on the emission wavelength. From 0.005 g to 0.025 g, the fluorescence intensity gradually increased with an increase in the amount of raw materials, which indicates that the raw material for forming CDs had not reached saturation. However, from 0.025 g to 0.045 g, the amount of raw materials for the reaction saturated. The presence of the raw material that did not participate in the reaction causes a partial fluorescence quenching, resulting in a decrease in the fluorescence intensity. The optimal amount was thus set at 0.025 g.

#### 3.1.3 The impact of temperature and time

The reaction temperature and time are critical factors that impact the optical properties of the as-prepared CDs.^33^ With other parameters fixed, the impact of the reaction temperature was investigated. The fluorescence increased as the temperature rose from 150 °C to 170 °C, and it decreased when the temperature kept rising to 190 °C (Fig. S3a†). Similarly, the fluorescence increased as the time increased from 3.5 to 5.5 h, and decreased when the time increased to 6 h (Fig. S3b†). We finally determined the optimal conditions as 170 °C and 5.5 h.

### 3.2 Separation

**Fig. 2:**
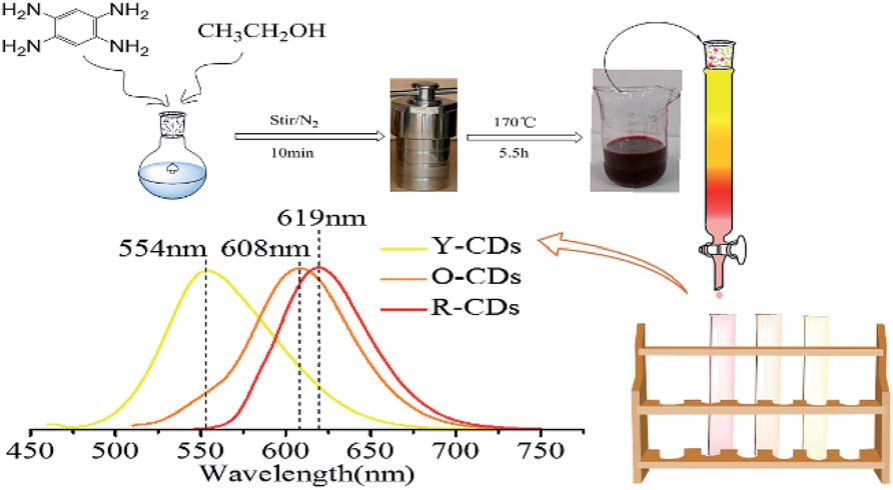
One-pot synthesis and purification route for CD-tetra. Corresponding PL emission spectra of R-, O- and Y-CDs, with maxima at 619, 608 and 554 nm, respectively.

Since previous literatures have reported on the luminescence mechanism of CDs through the separation process, the same research was carried out through the separation herein. MCI Gel chromatography was adopted because of its high separation efficiency, easy operation, large sample loading, and environmentally friendly solvents. As shown in Fig. 2, with CD-tetra as the precursor for separation, MCI Gel was pretreated with methanol for 30 min, and then filled into a column for separation (the MCI Gel powder mass at 20 g, filling height at 20 cm, loading product at 0.1 g). Through gradient elution (methanol and water as eluents, methanol 30–100%), red, orange and yellow-emitting CDs were acquired and marked as R-CDs, O-CDs and Y-CDs, respectively. The acquired CD solutions were then dried into solids for characterization.

### 3.3 Characterization

#### 3.3.1 Fluorescence and UV-Vis spectra

**Fig. 3:**
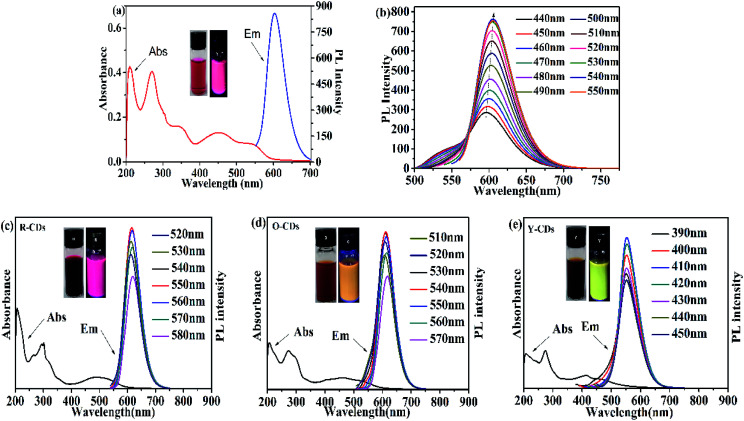
(a) UV-Vis spectra and emission spectra of CD-tetra. (b) Emission spectra of CD-tetra excited from 440 nm to 550 nm. (c) UV-Vis spectra and emission spectra of R-CDs. (d) UV-Vis spectrum and emission spectrum of O-CDs. (e) UV-Vis spectrum and emission spectrum of Y-CDs; insets are under ambient lighting (left) and 365 nm UV light (right), and the solvent used for all samples is ethanol.

Fluorescence and UV-Vis spectroscopy were used to investigate the optical properties of the acquired CDs before the separation procedure (Fig. 3a). The absorption peak at 210 nm in the UV-Vis spectrum of CD-tetra originates from the inherent state π–π* transition of the aromatic sp^2^ orbitals (C=C and C=N); the absorption peak at 270 nm originates from the defect state n–π* transition, which mainly includes the oxygen-containing functional groups C–O and C=O; and the peak at 540 nm is usually referred to as the transition of the surface groups. The fluorescence spectrum shows an emission peak at 605 nm under an excitation wavelength of 540 nm. The peak was symmetric with a narrow half bandwidth. The fluorescence intensity increases when the excitation wavelength shifts from 490 nm to 540 nm, and reaches a maximum at 540 nm (Fig. 3b). This shows that CD-tetra exhibited excitation wavelength dependence, where the emission peak redshifts with an increase in the excitation wavelength. We explored the impact of the pH value upon the stability of CD-tetra. The PBS buffer solution was prepared by NaH_2_PO_4_ and Na_2_HPO_4_. Fig. S4† shows the effect of pH (3–13) on the fluorescence property of CD-tetra. The fluorescence intensity of CD-tetra is strong in the pH range of 3 to 5, and the fluorescence intensity decreases with an increase in the pH value.

Fig. 3c, d and e are the fluorescence and UV-Vis spectra of the R-CDs, O-CDs and Y-CDs acquired from the separation of CD-tetra, with optimal excitation wavelengths at 550 nm, 540 nm and 410 nm, and corresponding emission wavelengths at 619 nm, 608 nm and 554 nm, respectively. The UV-Vis spectra of the three CDs exhibit the same absorption peaks at 207 nm and 270 nm, and their maximum absorption peaks at 492, 462 and 414 nm, respectively. Their emission peaks red-shift with the increase in the absorption wavelengths, which might be the reason for their different fluorescence colors. The emission wavelength positions of the R-CDs, O-CDs, and Y-CDs are different and essentially have no excitation wavelength dependence, indicating that the three CDs are more uniform in their optical properties than CD-tetra before separation. We believe that CD-tetra has an excitation wavelength dependence due to a mixture of these three CDs. Different luminescent CDs have different dominances at different excitation wavelengths for the CD-tetra mixture. As the excitation wavelength increases, the CDs with longer emission wavelengths become dominant. Thus, the emission wavelengths redshift as the excitation wavelength increases.

#### 3.3.2 TEM and XRD

**Fig. 4:**
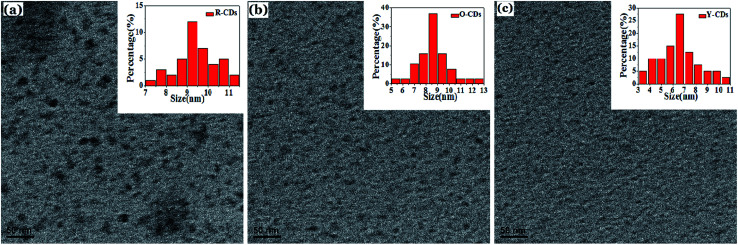
(a), (b) and (c) are the TEM images of the R-CDs, O-CDs and Y-CDs, respectively. The insets of particle size distribution of R-CDs, O-CDs and Y-CDs are illustrated, respectively.

Transmission electron microscopy (TEM) was used to study the morphology and particle size of the as-prepared products. As shown in Fig. 4, all three samples show a uniform particle size distribution and most particles are amorphous carbon particles without a crystal lattice structure. The average particle sizes of the R-CDs, O-CDs and Y-CDs are 9.39, 8.60 and 6.50 nm, respectively. The larger particle sizes would lead to a longer emission wavelength, which is one of the reasons for their fluorescence color differences. The XRD patterns of the three CDs (Fig. 4) show a broad peak in the range of 23–26° (2*θ*), further indicating highly disordered carbon atoms.^34,35^ This is consistent with the TEM results, in which no discernible lattice structures were observed. However, the peaks of the three CDs are at 23°, 25° and 26° (2*θ*), and the peak values decrease with the increase in the emission wavelengths.

#### 3.3.3 FTIR spectra

**Fig. 5:**
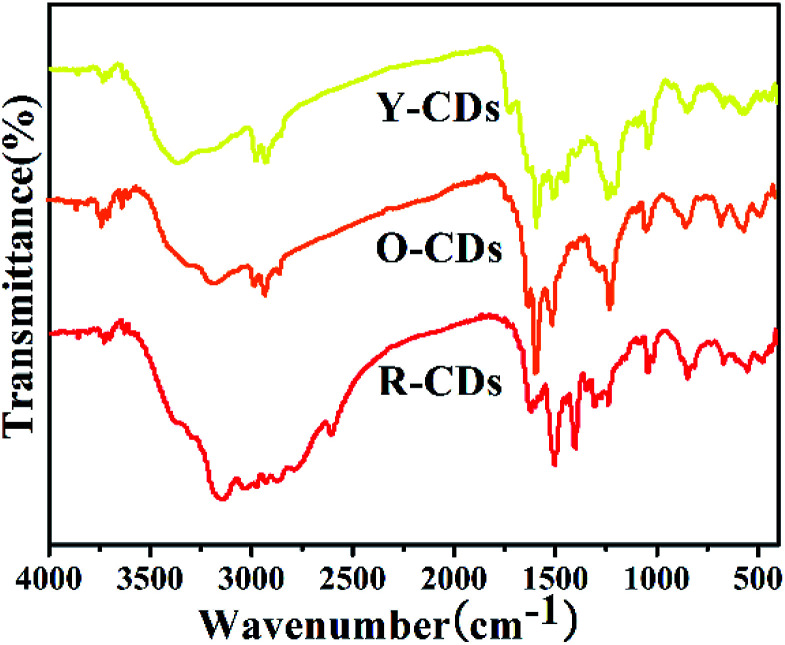
FTIR spectra of R-CDs, O-CDs and Y-CDs.

Fourier transform infrared spectroscopy (FTIR) was used to understand the sample components and consequently, their luminescence mechanism. As shown in Fig. 5, the peaks at 1633 cm^−1^, 1510 cm^−1^, 1430 cm^−1^, 1234 cm^−1^ and 1018 cm^−1^ show the existence of the C=O, C=C, C–N=, C–O and C–N groups, respectively; the peaks at 853 cm^−1^ and 664 cm^−1^ show the C–H bending vibration on the benzene ring; the peaks at 2928 cm^−1^ and 2839 cm^−1^ show the existence of =C–H. In addition, certain differences exist among the spectra. The existence of the N–H group was shown at 3149 cm^−1^ for R-CDs and O-CDs, but at 3369 cm^−1^ for Y-CDs. The spectrum of R-CDs shows more groups in the 2500–3500 cm^−1^ region than those of the other two CDs, illustrating that the rich surface groups are beneficial to the redshift in the CDs emission wavelength. Furthermore, the existence of aromatics rich in amino groups and the hydrophilic groups containing N–H and C–O endow the as-prepared CDs with excellent solubility in water, methanol and ethanol.

#### 3.3.4 XPS spectrum

X-ray photoelectron spectroscopy (XPS) was used to analyze the elemental components of the as-prepared CD samples. As shown in Fig. S6,† the spectra of the three CDs all show characteristic absorption peaks at 285, 399 and 531 eV, illustrating the existence of C1s, N1s and O1s,^21^ respectively. A comparison of the three spectra shows that the peaks of C1s, N1s and O1s are at the same location (Fig. S7†). The band can be differentiated into three peaks that correspond to carbon sp^2^ (C=C, 284.6 eV), carbon sp^3^ (C–O/C–N, 286.1 eV) and the carbonyl carbon (C=O, 287.9 eV). The O1s band has two peaks at 531.6 and 533.0 eV, corresponding C=O and C–O groups. The N1s band can be differentiated into three peaks at 398.4, 399.1 and 400.2 eV, representing the pyridine N, amino N and pyrrole N, respectively. These results prove that the as-prepared CDs contain a lot of nitrogen-containing fused ring structures, and the functional groups shown in the XPS spectra are in accordance with those in the FTIR spectrum. It is worth mentioning that although the three CD samples showed the same functional groups in the XPS spectrum, their elemental content differed. As shown in Table 2, from R-CDs, O-CDs to Y-CDs, their oxygen element content gradually decrease with the increase in the emission wavelengths. The R-CDs showed the highest nitrogen content (16.38%) of the three, and the difference in the nitrogen content between the O-CDs and Y-CDs was negligible.

**Table 2:** XPS elemental analysis results of R-CDs, O-CDs and Y-CDs

Sample	C (mol%)	O (mol%)	N (mol%)
R-CDs	73.18	10.44	16.38
O-CDs	76.16	13.88	9.96
Y-CDs	71.98	18.00	10.02

#### 3.3.5 The synthesis and luminescence mechanism of CDs

**Fig. 6:**
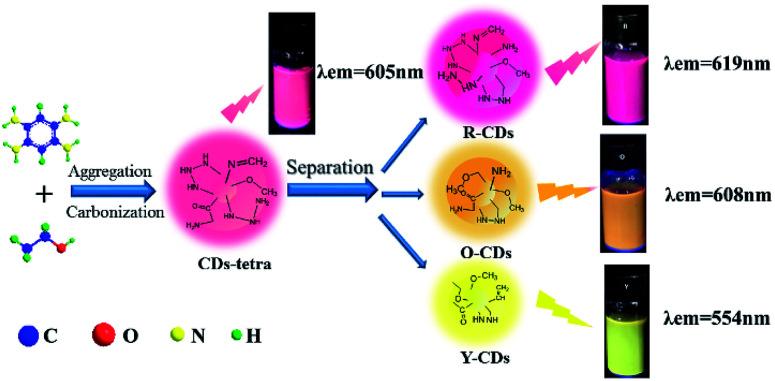
The possible synthesis and luminescence mechanism of CDs.

Through detailed investigation of the synthesis parameters and the process of separation and characterization, the synthesis and luminescence mechanism of the as-prepared CDs were studied. First, the selection of raw materials plays a critical role in the synthesis, which determines the emission wavelength of the CDs. For instance, Lin *et al.*^32^ used solvents to heat treat phenylenediamine with different replacement sites, and acquired three blue, green and red-emitting CDs. In our study, *O*-phenylenediamine, 1,2,4-benzenetriamine dihydrochloride and 1,2,4,5-benzenetetramine tetrahydrochloride were selected as a carbon source to prepare the CDs. Because 1,2,4,5-benzenetetramine tetrahydrochloride has more amino groups and a high nitrogen content, it therefore tends to produce longer wavelength CDs. The amount of raw materials would also impact the fluorescence property of the as-prepared CDs. Liang *et al.*^36^ prepared green, yellow and red-emitting CDs by adjusting the amount of *O*-phenylenediamine or *P*-phenylenediamine. In their experiments, the amount of phenylenediamine had a great impact upon the emission wavelength of the as-prepared CDs. In our experiments, the amount of raw material would only influence the fluorescence intensity rather than the emission wavelength of the as-prepared CDs. In addition, the fluorescence intensity of the CDs is also affected by temperature and time. As shown in Fig. 6, under a proper temperature and reaction time, the 1,2,4,5-benzenetetramine tetrahydrochloride and ethanol solution would form the red-emitting CD-tetra through a carbonization and aggregation process. To better understand the luminescence mechanism of the as-prepared CDs, separation was used to analyze the acquired product. With the MCI Gel filler as the stationary phase, and ethanol and water as the mobile phase, the R-CDs, O-CDs and Y-CDs were acquired by gradually reducing the polarity of the mobile phase. The fluorescence spectrum showed that CD-tetra possessed a certain excitation wavelength dependency before separation, but these three acquired CDs did not show such dependency. The reasons for such dependency reported that the CDs itself would emit different colored luminescence under different excitation wavelengths.^37^ In our study, it was discovered that the dependency of the CD-tetra originates from the various products. In the UV-Vis spectrum, the maximum absorption peaks of the three separated CDs redshift with an increase in the emission peak.

Three proposed mechanisms have been shown to be reasonable, involving the quantum confinement effect or the conjugated π-domain (determined by the carbon core),^32^ surface state (determined by the carbon skeleton or the attached chemical groups),^38^ or the molecular state (determined by the surface or internal fluorescence molecules of CDs).^39^ Ding *et al.*^21^ proved that the surface state is the key factor that determines the fluorescence change rather than the quantum size effect, and the increases in both the carboxyl content and oxidation degree on the CDs surface would cause the redshift of the emission wavelength. The raw materials employed in our synthesis are aromatic compounds with amino groups using ethanol as the solvent. Based on the characterization results from TEM, FTIR and XPS of the separated red, orange and yellow-emitting CDs, it was discovered that the optical properties of the as-prepared CDs are determined by both the quantum size effect and surface state. The emission wavelength of the CDs redshifts with an increase in the CD particle size, and an increase in the elemental nitrogen content would also cause the redshift. Since the R-CDs contain more nitrogen, there would be more amino groups on the surface of those CDs. This leads to a decrease in the energy gap between the highest occupied molecular orbit (HOMO) and lowest unoccupied molecular orbit (LUMO),^40^ and thus a longer emission wavelength than that of the O-CDs and Y-CDs.

### 3.4 Fluorescent detection of MB

**Fig. 7:**
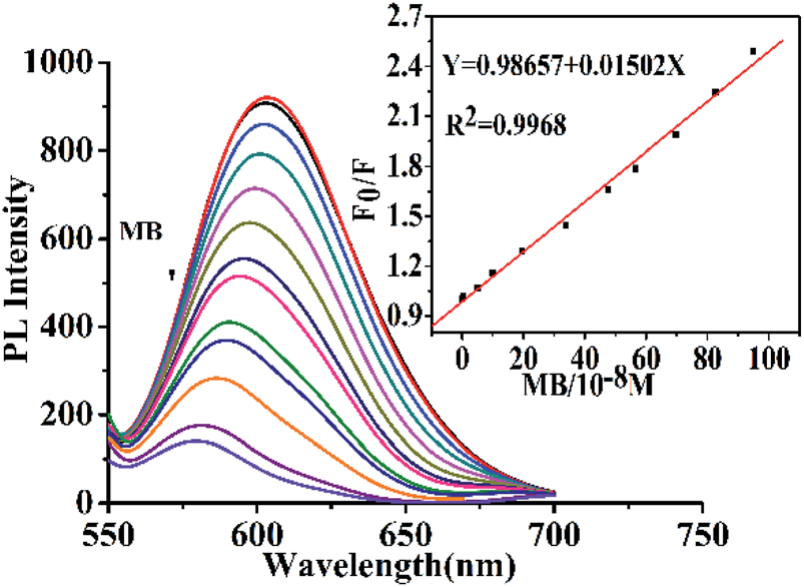
Emission spectra of CD-tetra after adding MB (from top to bottom: [MB] = 0, 0.05, 0.498, 0.99, 1.96, 3.38, 4.70, 5.60, 6.90, 8.20, 9.50, 13.0, 22.7, 27.1 μM). Inset: The standard curve of the relationship between *F*_0_/*F*.

The PL quenching effect of ions and dyes on CD-tetra was investigated. Different samples, including Co^2+^, Zn^2+^, Fe^3+^, Ca^2+^, Cu^2+^, Cd^2+^, Hg^2+^, SDI (concentration: 100 μM) and MR (concentration: 50 μM) and MB (concentration: 22.7 μM) were each reacted with a CD-tetra solution. As shown in Fig. S8,† the *F*_0_/*F* of CD-tetra could be distinctly quenched only with the addition of MB, and there was a small change in *F*_0_/*F* for the CD-tetra after adding metal ions and other dyes (*F*_0_ and *F* are the fluorescence intensity before and after the addition of detected substances). This result revealed that CD-tetra was selective toward MB over the other metal ions and dyes and could be developed as an efficient fluorescence sensor for MB. Fig. 7 shows the quenching effect of the fluorescence intensity towards MB. The fluorescence intensity of CD-tetra decreases gradually with the increase in the MB concentration. The *F*_0_/*F* values were obtained for CD-tetra treated with a concentration gradient of MB. A good linear correlation (*R*^2^ = 0.9968) was exhibited over the concentration range of 0.05–9.5 μM. According to the IUPAC standard, the LOD was taken as 3× standard deviation/slope and was calculated as 10 nM.^41^ For the fluorescence quenching mechanism, the UV-Vis absorption spectra of MB and the emission spectra of CDs-tetra are compared (Fig. S9†). The UV-Vis absorption spectrum of MB shows a broad absorption peak at 434–700 nm, which greatly overlaps with the emission spectra of CD-tetra, which enables the effective screening of emission from CD-tetra by MB. This indicates that the fluorescence of the CD-tetra is quenched by MB due to the internal filtration effect.

## 4. Conclusions

This study, for the first time, reported the fast and simple synthesis of red-emitting CD-tetra *via* a one-step solvothermal route with 1,2,4,5-benzenetetramine tetrahydrochloride as the carbon source and absolute ethanol as the solvent. Three aniline compounds with different amino numbers were chosen to prepare the CDs under the same synthesis parameters, and the experiments showed that CD-tetra prepared by 1,2,4,5-benzenetetramine tetrahydrochloride with more amino groups exhibited excellent optical properties. The experiment originated from the variations found in the products. The three separated CD products exhibited obvious differences in their particle sizes and elemental content. Furthermore, CD-tetra could be used as a probe for the fluorescence detection of MB in the range of 0.05–9.5 μM and the LOD of CDs-tetra is 10 nM.

## Conflicts of interest

There are no conflicts to declare.

## Supplementary Material

RA-009-C9RA05570C-s001
