# Supplementary material for: One-step synthesis of red-emitting carbon dots via a solvothermal method and its application in the detection of methylene blue
Source: RSC Adv. 2019 Sep 18;9(51):29533–40. doi: 10.1039/c9ra05570c (PMC9072023; doi:10.1039/c9ra05570c)
Supplement: RA-009-C9RA05570C-s001 [file RA-009-C9RA05570C-s001.pdf]

Electronic Supplementary Material (ESI) for RSC Advances.
This journal is © The Royal Society of Chemistry 2019

Supplementary material

One-step synthesis of red-emitting carbon dots *via* a solvothermal method and its application in the detection of methylene blue

Dan Zhao, * Xuemei Liu, Chunjin Wei, Yimo Qu, Xincai Xiao, Han Cheng

School of Pharmaceutical Sciences, South-Central University for Nationalities, Wuhan 430074, P.R. China

Corresponding Author: * Dr. Dan Zhao, Email: wqzhdpai@163.com

Address:Minzu Avenue#182, School of Pharmaceutical Sciences, South-Central University for Nationalities,Wuhan 430074, P.R. China; Tel:18062084690; Fax:027-67841196

CDs-tetra λ_{ex} =495 nm in EtOH   Rhodamine B λ_{ex} =495 nm in EtOH

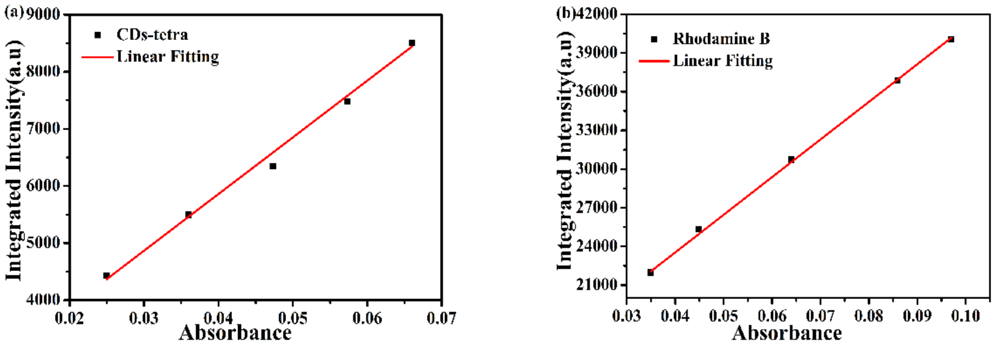

Fig.S1 Plots of integrated PL intensity of CDs-tetra and rhodamine B (referenced dye) as a functionof optical absorbance at 495nm.

Table S1 Data related to quantum yield calculation

	CDs-tetra					Rhodamine B				
Abs	0.0250	0.0360	0.0473	0.0573	0.0660	0.0350	0.0448	0.0640	0.0859	0.0970
Integrated PL	4431	5495	6351	7477	8501	21979	25320	30728	36876	40076
Slope	9.76×10^4					2.88×10^5				
QY	30.2%					56%				

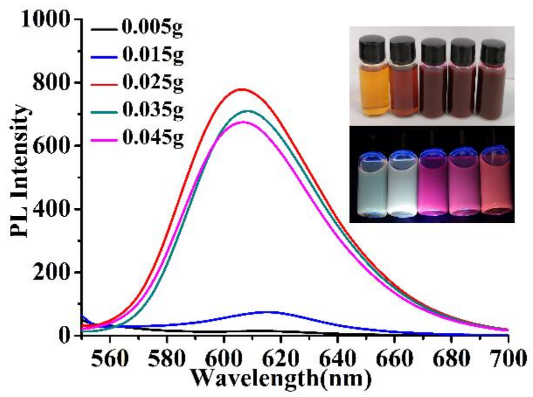

Fig.S2 Fluorescence spectra of CDs-tetra synthesized from different dosages of raw materials. Insets: photos of CDs solution fluorescent lamp (top) and 365 nm ultraviolet lamp (bottom) synthesized from left to right with 0.005 g, 0.015 g, 0.025 g, 0.035 g and 0.045 g raw materials, respectively.

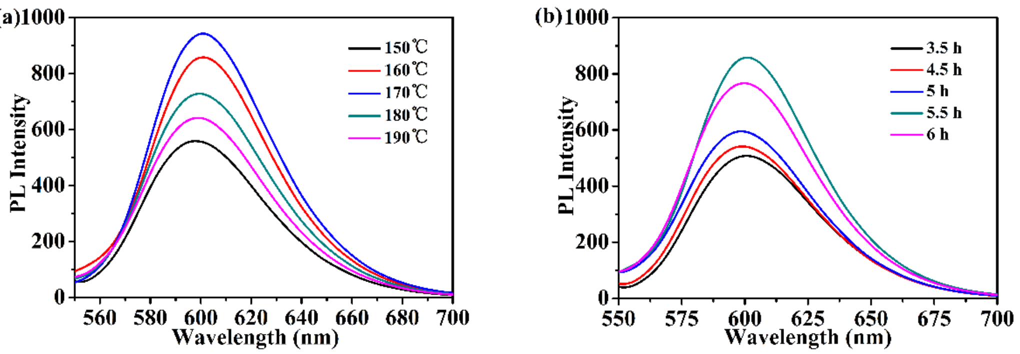

Fig.S3 (a) Effect of reaction temperature on synthesis of CDs-tetra (b) Effect of reaction time on synthesis of CDs-tetra.

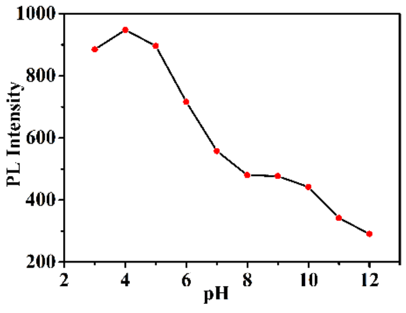

Fig.S4 PL Intensity at the peak position of CDs-tetra with different pH values.

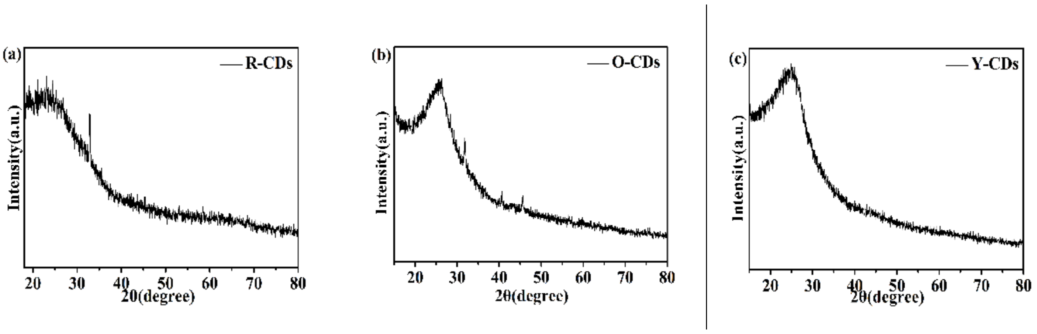

Fig.S5 XRD pattern of the R-CDs(a), O-CDs(b) and Y-CDs(c).

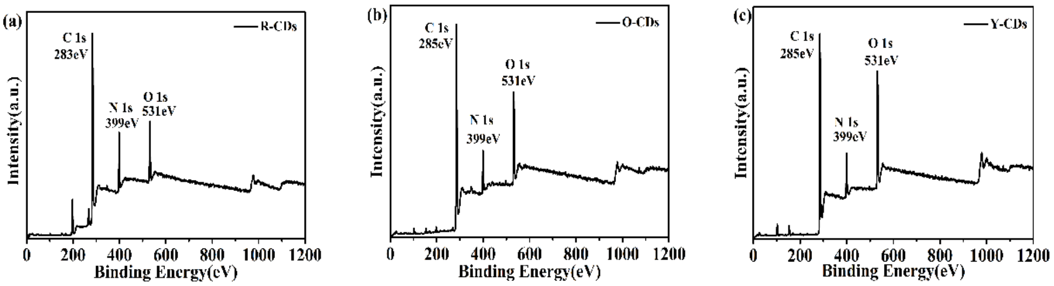

Fig.S6 Full XPS spectra of R-CDs(a), O-CDs(b) and Y-CDs(c).

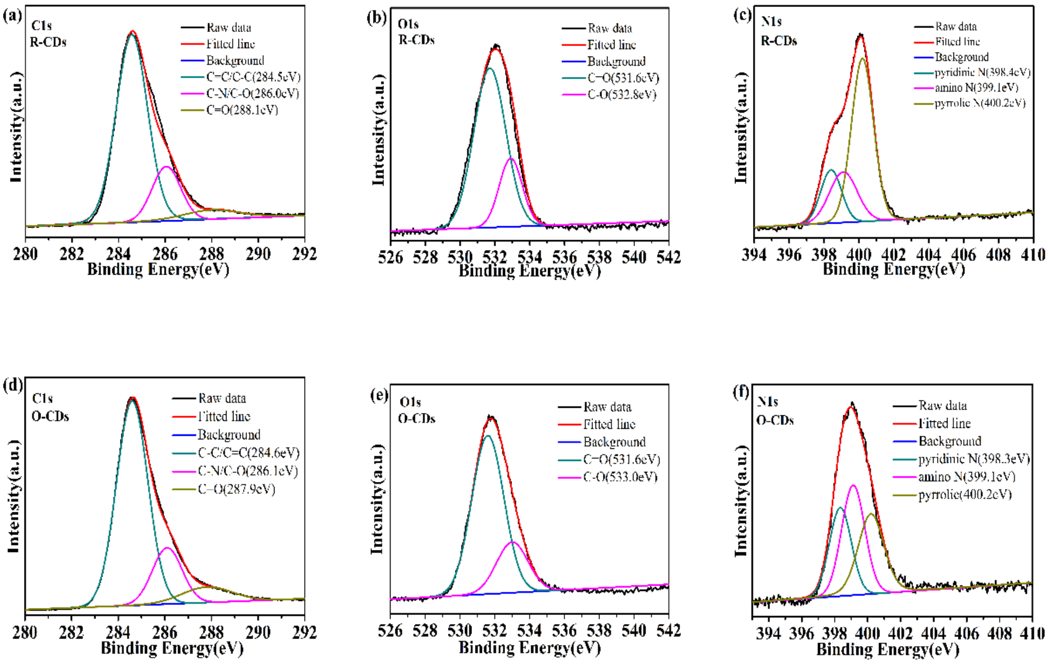

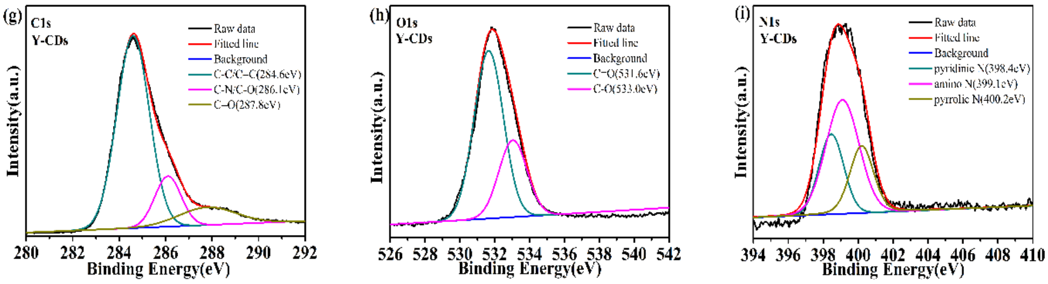

Fig.S7 C 1s, O 1s and N 1s spectra of R-CDs (a, b and c), O-CDs (d, e and f) and Y-CDs (g, h and i).

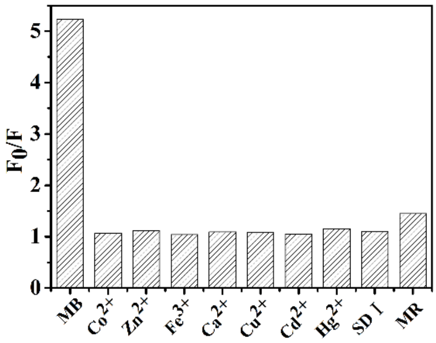

Fig.S8 Sensitivity investigation of the CDs-tetra for MB detection (MB :22.7μM, MR:50μM and other samples concentration:100μM).

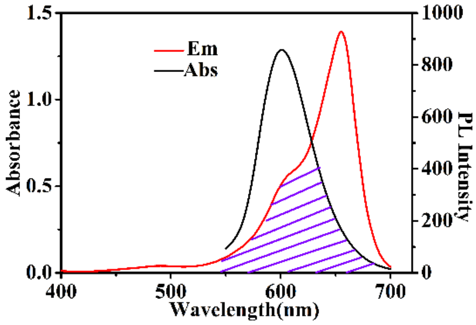

Fig.S9 The overlap between absorption spectrum of MB and the emission spectra of CDs-tetra.
